# Anticancer Potential of Valencia Peanut (*Arachis hypogaea* L.) Skin Extract against Cervical Cancer Cells In Vitro and in Nude Mouse Xenograft Models

**DOI:** 10.3390/foods13152354

**Published:** 2024-07-26

**Authors:** Jarckrit Jeeunngoi, Gulsiri Senawong, Sanun Jogloy, Jeerati Prompipak, Arunta Samankul, Suppawit Utaiwat, Khanutsanan Woranam, Banchob Sripa, Thanaset Senawong

**Affiliations:** 1 Department of Biochemistry, Faculty of Science, Khon Kaen University, Khon Kaen 40002, Thailand; jarckrit@kkumail.com (J.J.); gulsiri@kku.ac.th (G.S.); jeerati.ppk@gmail.com (J.P.); s_arunta@kkumail.com (A.S.); suppawitu@gmail.com (S.U.); k.woranam@gmail.com (K.W.); 2 Department of Plant Science and Agricultural Resources, Faculty of Agriculture, Khon Kaen University, Khon Kaen 40002, Thailand; sanun@kku.ac.th; 3 WHO Collaborating Centre for Research and Control of Opisthorchiasis (Southeast Asian Liver Fluke Disease), Tropical Disease Research Center, Faculty of Medicine, Khon Kaen University, Khon Kaen 40002, Thailand; banchob@kku.ac.th

**Keywords:** cervical cancer, apoptosis, *Arachis hypogaea* L., drug combination, xenograft model, peanut skin extract

## Abstract

This study investigated the impact of Valencia KK4-type peanut skin ethanolic extract (KK4-PSE) combined with cisplatin or 5-fluorouracil (5-FU) on HeLa cells in vitro and in xenograft models. At exposure times of 24, 48 and 72 h, KK4-PSE inhibited the growth of HeLa cells with a half maximal inhibitory concentration (IC_50_) of 79.43 ± 0.54, 55.55 ± 1.57 and 41.32 ± 0.74 µg/mL, respectively. Drug interactions evaluated by the Chou–Talalay method demonstrated that KK4-PSE enhanced antiproliferative activity of 5-FU against HeLa cells with combination index (CI) values of 0.49 (48 h) and 0.60 (72 h), indicating a synergistic effect, while KK4-PSE combined with cisplatin exhibited an additive effect (CI = 1.02) at 72 h, and an antagonistic effect at 24 and 48 h exposures (CI = 1.12 and 1.18, respectively). In nude mouse xenograft models, the combination of 5-FU and KK4-PSE markedly reduced HeLa tumor weights compared with the control and single agent treatments groups. The combination of KK4-PSE and 5-FU achieved greater tumor growth inhibition than that of the KK4-PSE–cisplatin combination. KK4-PSE mitigated hepatotoxicity induced by both cisplatin and 5-FU in nude mice. The spleen hyaloserositis was significantly reduced in the combination treatment of 5-FU and KK4-PSE. These results suggest that KK4-PSE has the potential to limit cervical cancer cell proliferation while reducing the toxicity of cisplatin and 5-FU.

## 1. Introduction

Cervical cancer is the fourth most common cancer in women regarding both incidence and mortality, with an estimated 660,000 new cases and 350,000 deaths worldwide in 2022 [1]. Cervical cancer is the most common cancer type in 25 countries and the leading cause of cancer death in 37 countries [1,2]. In most cases, co-administration of cisplatin and paclitaxel is the main procedure treatment for advanced cervical cancer. However, the response rate of the combined drugs is 29.1–67% in patients who had recurrence after receiving combination treatment [3]. Despite the improvement following cisplatin combination therapy, the 5-year survival rate of patients with cervical cancer remains low [4]. Furthermore, tumor recurrence and the development of chemotherapy drug resistance are virtually unavoidable and are a major cause of less effective treatment [5]. As a result, there is an immediate requirement of aggressive systemic therapies that use a single drug or a combination of drugs [6]. However, the rising price and adversity of chemotherapy drugs cause a challenge for patients in developing countries. Therefore, alternative herbal supplements for cancer treatment remain an unavoidable option [7].

Chemotherapy drug resistance in cervical cancer remains a critical obstacle to effective practical therapy. It is important to identify pharmacologically safe agents capable of enhancing conventional therapy while reducing side effects from chemotherapy drugs. Polyphenol agents appear to have the potential to solve this problem, as several studies have reported that polyphenol agents possess antitumor activity against various types of cancer [8]. “Polyphenol compounds” are known as one of the potential natural product groups in cancer prevention [9]. The main anticancer effects of these compounds are associated with their antioxidant and anti-inflammatory properties, which involve multiple mechanisms of action [10]. To overcome cancer resistance, much effort has been concentrated on the development of anticancer drugs based on natural products with a diverse range of anticancer properties through multiple mechanisms. 

Peanut (*Arachis hypogaea* L.) seed skin (testa) has long been considered as an important source of polyphenols. Polyphenol compounds from peanut testa, particularly procyanidins, demonstrated remarkable activity against a variety of cancer cell lines [11]. According to our previous study, Valencia-type peanut skin extracts exhibited antitumor activity against several human carcinoma cell lines (breast, cervical, colon, and liver) in a dose- and time-dependent manner [12]. The antitumor activity of Valencia-type peanut skin extract is associated with a variety of mechanisms, including the activation of caspases (cysteinyl aspartate-specific proteases), the production of reactive oxygen species (ROS), and the inactivation of histone deacetylases (HDACs) [13,14]. KK4-PSE inhibited the growth of human cervical adenocarcinoma (HeLa) cell line by inhibiting the HDAC enzyme and inducing cell apoptosis [12]. Peanuts are a valuable source of biological function as well as a potential source of natural polyphenol compounds [15]. Peanut skin procyanidins (PSP) and their derivatives significantly inhibited prostate cancer via apoptotic cell death induction and cell cycle arrest mechanisms [16]. Peanut skins contain resveratrol, known as the naturally occurring phytoalexin that peanuts produce in response to stress, possessing anticancer properties [17]. We previously demonstrated that in vitro, peanut skin extracts from Valencia genotypes (ICG15042 and KK4) with HDAC inhibitory activity suppressed the proliferation of cancer cells [12]. Our findings are in accordance with previous publications that link HDAC inhibitory activity of the compounds to the inhibition of a tumor growth [18,19]. Peanut skin extract possessing HDAC inhibitory activity suppressed activity of HDAC enzymes in the cancer cells causing an accumulation of acetylated forms of histone proteins leading to apoptotic cancer cell death [12]. In addition, the phenolic acids of Valencia KK4-type peanut skin extract analyzed by HPLC include *p*-coumaric, vanillic, ferulic, *p*-hydroxybenzoic, sinapinic, and syringic acids [12]. KK4-type exhibited greater amounts of natural HDAC inhibitors (*p*-coumaric, ferulic, and sinapinic acids) than that of ICG15042-type [12]. These peanut phenolic acids possessing HDAC inhibitory activity demonstrated antiproliferative activity against a cervical cancer cell line (HeLa cells) in a dose- and time-dependent manner [20]. The phenolic acids, *p*-coumaric and vanillic acids, were the two most abundant compounds among all identified phenolic acids in KK4-type peanut skin; however, some HPLC peaks in the chromatogram of the peanut skin extract have yet to be identified [21]. 

Valencia KK4-type peanut skin ethanolic extract (KK4-PSE) exhibited anticancer activity against HeLa cervical cancer cells in vitro [12]. However, its anticancer activity in vivo and its interaction with current anticancer drugs, such as cisplatin and 5-fluorouracil (5-FU), have not yet been explored. Based on the findings that ethanolic extracts of Thai noni juice (TNJ) products and *Tiliacora triandra* leaf powder possessing HDAC inhibitory activity enhance the anticancer effect against human cholangiocarcinoma (CCA) cells and reduce toxicity of 5-FU, cisplatin and gemcitabine in nude mouse xenograft models [22,23], we therefore hypothesize that KK4-PSE would enhance anticancer activity of cisplatin or 5-FU against cervical cancer cells both in vitro and in nude mouse xenograft models. In the present study, we explored the anticancer potential of KK4-PSE to inhibit cervical cancer cell growth both in single and combination drug treatments with cisplatin or 5-FU in vitro (HeLa cervical cancer cells) and in vivo (nude mouse xenografts). The mechanisms underlying the synergistic drug interactions were also investigated. The results from this study may help researchers better understand and develop Valencia KK4-type peanut skin extract as a potential supportive agent during chemotherapy and/or chemoprevention program.

## 2. Materials and Methods

### 2.1. Materials and Reagents 

RPMI-1640 medium was obtained from Gibco-BRL (Gaithersburg, MD, USA), whereas fetal bovine serum (FBS) was purchased from Cytiva (Kremplstrasse, Pasching, Austria). Propidium iodide (PI) and 3-(4,5-dimethylthiazol-2-yl)-2,5-diphenyltetrazolium bromide (MTT) were purchased from Sigma-Aldrich Corporation (St. Louis, MO, USA), whereas the Annexin V-FITC was obtained from Biolegend (San Diego, CA, USA). Cisplatin and 5-fluorouracil (5-FU) were purchased from Sigma-Aldrich Corporation (St. Louis, MO, USA) and PanReac Applichem (Castellar del Valles, Spain), respectively. The antibodies against p21 (2946), p53 (2524), Bcl-2 (2870), Bax (2772), pERK1/2 (9107), ERK1/2 (4377), acetyl-histone H3 (9671), and CDK4 (12790) were obtained from Cell Signaling Technology (Beverly, MA, USA). The anti-cyclin B1 (GNS1) antibody was purchased from Santa Cruz Biotechnology (Dallas, TX, USA).

### 2.2. Cell Culture and Nude Mouse Xenograft Models 

HeLa and Vero cell lines, obtained from the American Type Culture Collection (ATCC, Manassas, VA, USA) in 2010, were maintained in culture in the Department of Biochemistry, Faculty of Science, Khon Kaen University, Thailand. The immortalized noncancer Vero cell line was used as a control in the antiproliferation assay due to its non-tumorigenic origin, and its sensitivity and reliability in detecting cytotoxic effects, thus facilitating the assessment of potential toxic effects on cellular systems. HeLa and Vero cells were cultured in RPMI-1640 medium supplemented with 10% fetal bovine serum (FBS), 100 U/mL penicillin, and 100 µg/mL streptomycin (all from Gibco, New York, NY, USA). The cultures were maintained at 37 °C in a humidified atmosphere containing 5% CO_2_. Only exponentially growing cells were used for all subsequent experiments. Female BALB/CAJcl-Nu/Nu mice (4–6 weeks old, weighing 25–30 g) were procured from Nomura Siam International (Bangkok, Thailand). The mice were housed in individual ventilated cages (IVCs) at 23 ± 2 °C, with humidity maintained at 30–60% and a 12-h light/dark cycle (350–400 Lux). All animal procedures were conducted at the Northeast Laboratory Animal Center, Khon Kaen University. 

### 2.3. Preparation of KK4-PSE 

Valencia KK4-type peanuts were harvested from a field crop grown at Khon Kaen University’s Field Crop Research Station, Thailand, during the 2019 season (October 2018 to February 2019). Seed skins were separated, dried in a hot air oven at 60 °C for 6 h, then powdered and stored under sterile conditions in plastic bags at 4 °C. For extraction, 1 g of peanut skin powder was mixed with absolute ethanol at a 1:40 (*w*/*v*) ratio and stirred at room temperature for 48 h. Ethanol was used as a solvent because of its relatively low toxicity and high extraction yield of phenolic compounds. The resulting supernatant was filtered through a Whatman No. 4 filter paper and concentrated by evaporation. The extract was subsequently stored at −20 °C until use.

### 2.4. Cell Viability Assay 

Cell proliferation assay was performed using the MTT Assay. Cells were seeded into 96-well plates at a density of 8 × $10^{3}$ cells/well and incubated at 37 °C for 24 h. The cells were then treated for various periods of time (24, 48 and 72 h) with varying concentrations of KK4-PSE alone or in combination with a sub-toxic dose of cisplatin or 5-FU drug at IC_20_ concentration (the concentrations required to inhibit 20% of cell growth) and a vehicle control (0.5% DMSO:EtOH, *v*/*v*). At a specified exposure time, the culture media were removed, and each well was filled with a fresh medium containing 1.2 mM MTT solution. After incubation at 37 °C for 2 h, DMSO (100 µL/well) was added and incubated for 15 min at room temperature. The absorbance (A) of dissolved formazan was measured at 570 nm using a Spectramax M5 microplate fluorometer (Molecular Devices Cooperation, Sunnyvale, CA, USA), and the optical density (O.D.) at 655 nm was measured to subtract the optical density of cellular debris at 570 nm. The experiments were performed at least three times.

$$\%Cell\;viability=\frac{A570\;Sample-O.D.655\;Sample}{A570\;Control-O.D.655\;Control}\times 100.$$

### 2.5. Drug Interaction Determination 

The CI value was calculated according to the median-effect principle to estimate the interactions between KK4-PSE and the two chemotherapy drugs [24,25]. The CI values for 50% growth inhibition were calculated using the following equation:

$$CI=\left(\frac{D1}{Dx1}\right)+\left(\frac{D2}{Dx2}\right)+\alpha \frac{D1D2}{Dx1Dx2}$$

where D1 is a dose of drug 1 (cisplatin or 5-FU) in a combination treatment with drug 2 (KK4-PSE) to produce 50% cell viability; Dx1 is a dose of drug 1 in a single treatment to produce 50% cell viability; D2 is a dose of drug 2 in a combination treatment with drug 1 to produce 50% cell viability; Dx2 is a dose of drug 2 in a single treatment to produce 50% cell viability; and α = 1 for mutually non-exclusive modes of drug action. 

The DRI indicates the extent of dose reduction (fold) of the combined dose tested compared to the dose in a single agent treatment. The DRI was calculated using the following equation:

$$DRI=\left(\frac{Dx}{D}\right),$$

where D is the dose of one drug combined with another drug to produce 50% cell viability; Dx is the dose of a drug in a single drug treatment to produce 50% cell viability. 

### 2.6. Cell Cycle Analysis 

Human cervical cancer (HeLa) cells (2.5 × 10^5^ cells/mL) were plated in a 5.5 5.5 cm culture dish and incubated for 24 h. The cells were then treated with various concentrations of KK4-PSE, either alone or in combination with the IC_20_ subtoxic dose of the chemotherapy drug (cisplatin or 5-FU). PI staining was performed as described previously. Synergistic concentrations of KK4-PSE and 5-FU were used to treat HeLa cells for 48 h. The analysis of DNA content in HeLa cells was conducted using PI staining and the BD FACSCanto II flow cytometer (Becton Dickinson, San Jose, CA, USA). Cell cycle distribution in sub-G1, G0/G1, S, and G2/M phases was determined using the BD FACSDiva software (version 8.0). 

### 2.7. Apoptosis Detection by Flow Cytometry 

Cellular apoptosis was evaluated using Vybrant Apoptosis Assay Kit #2, Molecular Probes, Invitrogen Corporation (Carlsbad, CA, USA) according to the manufacturer’s instructions. Apoptotic cells were stained with Annexin-V FITC and PI as previously described [26]. Briefly, HeLa cells were seeded at a density of 1 × 10^6^ cells/dish in a 5.5 cm culture dish and incubated for 24 h. Synergistic concentrations of KK4-PSE and 5-FU were used to treat HeLa cells for 48 h. Thereafter, cells were washed twice with cold PBS and resuspended in 1X ice-cold Annexin-binding buffer (100 µL/dish). Each tube was then loaded with 400 µL of 1X binding buffer and the cells were incubated with Annexin V-FITC and PI in the dark for 15 min at room temperature. To evaluate the effect of treatment on cell death, apoptotic cells were identified by Annexin V/PI staining and analyzed using the BD FACSDiva software (version 8.0). Specifically, the analysis quantified the percentage of Annexin V-positive and PI-negative cells, representing early apoptosis. 

### 2.8. Western Blot Analysis 

HeLa cells were plated at a density of 1 ×10^6^ cells/dish in a 5.5 cm culture dish. Synergistic concentrations of KK4-PSE and 5-FU were used to treat the HeLa cells for 48 h. Total proteins were extracted using RIPA lysis buffer (Amresco, Solon, OH, USA) with a protease inhibitor cocktail and the protein concentration was determined using the Bio-Rad protein assay (Bio-Rad, Hercules, CA, USA). The protein bands were resolved by SDS-PAGE (12.5%) and transferred to the polyvinylidene fluoride (PVDF) membrane. After incubation for 1 h at room temperature in TBST containing 5% skim milk, the blot membrane was then incubated with primary antibodies overnight at 4 °C. The primary antibodies used for Western blotting analysis included anti-p21 (1:2000), anti-p53 (1:1000), anti-Bcl-2 (1:1000), anti-Bax (1:1000), anti-pERK1/2 (1:2000), anti-ERK1/2 (1:1000), anti-cyclin B (1:1000), anti-CDK4 (1:1000), and anti-acetyl-histone H3 (1:1000). The membrane was washed with TBST before being incubated with the corresponding horseradish peroxidase-conjugated secondary antibodies (Cell Signaling Technology, Danvers, MA, USA). The blots were then developed with a chemiluminescence reagent (Bio-Rad, CA, USA) and exposed to the X-ray film. 

### 2.9. Antitumor Activity of KK4-PSE in Nude Mouse Xenograft Models 

Female nude mice (BALB/cAJcl-Nu/Nu, 4–6 weeks old) were obtained from Nomura Siam International (Bangkok, Thailand) and maintained at Khon Kaen University’s Northeast Laboratory Animal Center. The animal experiments were approved by Khon Kaen University’s Institutional Animal Care and Use Committee (approval No. IACUC-KKU-20/62; date of registration 21 March 2019) and performed in accordance with guidelines established by the National Research Council of Thailand’s Ethical Principles and Guidelines for the Use of Animal in Scientific Purposes (License No. U1-00998-2558). The study was carried out in compliance with the ARRIVE guidelines. To obtain cervical cancer xenografts, HeLa cell suspensions (4 × 10^6^ cells in 100 µL) were subcutaneously injected into the right anterior lateral thoracic wall of each mouse [27]. After cell implantation for 14 days, mice were randomly divided into nine groups of five mice each. Mice in the experimental groups were given intraperitoneal injections of KK4-PSE at doses of 100 and 200 mg/kg body weight twice a day, cisplatin at a dose of 3 mg/kg body weight twice a day, 5-FU at a dose of 10 mg/kg body weight twice a day, and combinations of KK4-PSE and cisplatin or 5-FU. In the control group, mice were given an equal volume of normal saline solution. A digital vernier caliper was used to measure the tumor volume which was calculated every two days as the following formula:

$$Tumor\;volume=\frac{length\times {width}^{2}}{2}.$$

The tumor growth inhibition ratio (TGI, %) was determined using the following formula:

$$\%TGI=[\left(A-B\right)/A]\times 100,$$

where A was mean tumor weight of the vehicle control group and B was tumor weight of the treated group. 

### 2.10. Toxicological Evaluation in Nude Mouse Xenograft Models

Toxicities of the drugs on xenograft mice during treatments were assessed by monitoring body weight changes, organ weight, and histopathology of organs (liver, kidneys, and spleen). After 14 days of treatment, the mice were sacrificed, and the tumors and visceral organs were weighed before being fixed in 10% formalin solution for the further experiments. Additionally, a toxicity analysis was conducted through the assessment of body weight changes (%BWC) following the formula:

$$\%BWC=\frac{Final\;body\;weight\left(g\right)-Initial\;body\;weight\left(g\right)}{Initial\;body\;weight(g)}\times 100.$$

Following fixation in 10% formalin and subsequent dehydration and clearing in the tissue processor, the tumor, liver, kidney, and spleen tissues were paraffin embedded. Samples were sectioned at 5 μm using a Leica RM2255 Fully Automated Rotary Microtome (Leica Microsystems, Wetzlar, Germany) and mounted onto glass slides. Deparaffinization was achieved using xylene, followed by rehydration through a graded series of ethanol solutions (99%, 95%, and 70%) and rinsing in distilled water. The rehydrated tissue sections were then stained with hematoxylin and eosin (H&E) and examined under a light microscope (Eclipse E200, Nikon, Tokyo, Japan).

### 2.11. In Situ Apoptosis Detection 

The level of apoptosis within tissue sections was assessed using the terminal deoxyuridine nick-end labeling (TUNEL) assay. Following the protocol of the In Situ Cell Death Detection kit (Roche Applied Science, Mannheim, Germany), all specimens were sequentially fixed with 10% formaldehyde, dehydrated, paraffin-embedded, and sectioned into 5 µm-thick histological sections. After TUNEL staining, slides were counterstained with 10% hematoxylin, dehydrated, mounted, and visualized under a light microscope (Olympus CX31, Tokyo, Japan). TUNEL-positive cells were identified and quantified by counting the number of brown-stained (DAB-positive) cells per field image using the particle analysis tool in ImageJ Fiji software (version 1.53), adapted from the method described previously [28]. The data were then expressed as the average percentage of TUNEL-positive cells.

### 2.12. Statistical Analysis 

Statistical analyses were performed using SPSS 22.0 (IBM, Manassas, VA, USA). The data are presented as mean ± standard deviation (SD) and mean ± SEM (for independent experiments). Graphical representations were generated with GraphPad Prism 8.0 (GraphPad Software, La Jolla, CA, USA). One-way analysis of variance (ANOVA) was employed to assess statistically significant differences between the control and experimental groups, followed by Duncan’s post hoc test for pairwise comparisons. The *p* values < 0.05 were considered statistically significant. All experiments were performed in triplicate.

## 3. Results

### 3.1. Antiproliferative Activities of KK4-PSE, Cisplatin, and 5-Fluorouracil against Cervical Cancer Cells in Single-Agent Treatments 

**Figure 1:**
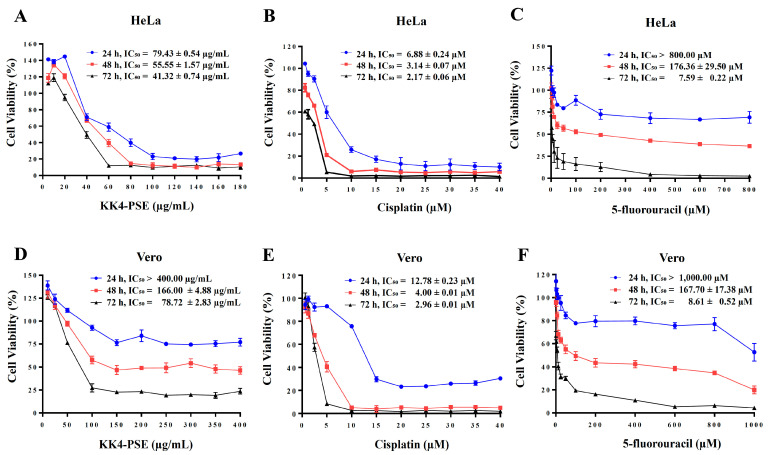
Antiproliferative effects of the single agent treatments of KK4-PSE, cisplatin, and 5-FU. Human cervical cancer HeLa cells (**A**–**C**) and non-cancer Vero cells (**D**–**F**) were treated with various concentrations of KK4-PSE or chemotherapeutic drugs (cisplatin, 5-FU) for 24, 48 and 72 h. The percentages of cell viability were calculated relative to the solvent control treatment (0.50% ethanol + 0.50% DMSO). Data shown were mean ± SEM from three independent experiments.

The antiproliferative effects of KK4-PSE and current chemotherapeutic agents (cisplatin and 5-FU) against cervical cancer cells were assessed by the MTT assay. KK4-PSE, cisplatin, and 5-fluorouracil suppressed proliferation of HeLa cells in a dose- and time-dependent manner as shown in Figure 1. At exposure times of 24, 48 and 72 h, KK4-PSE inhibited the growth of HeLa cervical cancer cells with a half maximal inhibitory concentration (IC_50_) of 79.43 ± 0.54, 55.55 ± 1.57 and 41.32 ± 0.74 µg/mL, respectively (Figure 1A), and showed less toxicity on the non-cancer cells (Vero cells) with IC_50_ values of >400, 166.00 ± 4.88 and 78.72 ± 2.83 µg/mL, respectively (Figure 1D). The current anti-cancer drug cisplatin significantly inhibited proliferation of HeLa (Figure 1B) and Vero (Figure 1E) cells at all exposure times, whereas 5-FU significantly inhibited proliferation of HeLa (Figure 1C) and Vero (Figure 1F) cells at exposure times of 48 and 72 h only. Cisplatin inhibited the growth of HeLa cells with IC_50_ values of 6.88 ± 0.24, 3.14 ± 0.07 and 2.17 ± 0.06 µM at exposure times of 24 h, 48 h and 72 h exposures, respectively, while 5-FU suppressed the growth of HeLa cells with IC_50_ values of >800, 176.36 ± 29.50 and 7.59 ± 0.22 µM at exposure times of 24, 48 and 72 h, respectively.

### 3.2. Antiproliferative Activities of KK4-PSE in Combination Treatments with Current Anticancer Drugs (Cisplatin and 5-FU) against Cervical Cancer Cells

The antiproliferative activities of KK4-PSE combined with subtoxic doses (IC_20_) of current chemotherapeutic drugs (cisplatin and 5-FU) against HeLa cells were determined. The IC_50_ values of KK4-PSE in combinations with subtoxic doses of cisplatin and 5-FU were shown in Table 1. The CI and DRI values were calculated to determine the type of drug interaction based on the median-effect principle of the Chou & Talalay method [19,20]. The combination treatments with KK4-PSE and 5-FU of cervical cancer cells at 48 and 72 h exposures gave CI values of 0.49 and 0.60, respectively, indicating a synergistic effect (Table 1). These synergistic effects at 48 and 72 h exposures resulted in dose reductions of 5.09- and 21.78-fold for 5-FU and 2.13- and 2.49-fold for the KK4-PSE, respectively. The combination treatments of KK4-PSE and cisplatin exhibited an additive effect (CI = 1.02) at 72 h exposure but showed a moderate antagonistic effect at 24 and 48 h exposures (CI = 1.12 and 1.18, respectively) in HeLa cells (Table 1). The additive effect of KK4-PSE and cisplatin at 72 h exposure in HeLa cells resulted in the greatest dose reduction (7.30-fold) for cisplatin. These findings suggest that treatment with KK4-PSE enhances the anticancer activity of 5-FU against cervical cancer cells more effectively than the combination with cisplatin.

### 3.3. Effect of the Combination Treatments of KK4-PSE and 5-FU or Cisplatin on Inductions of Cell Cycle Arrest and Apoptosis 

**Figure 2:**
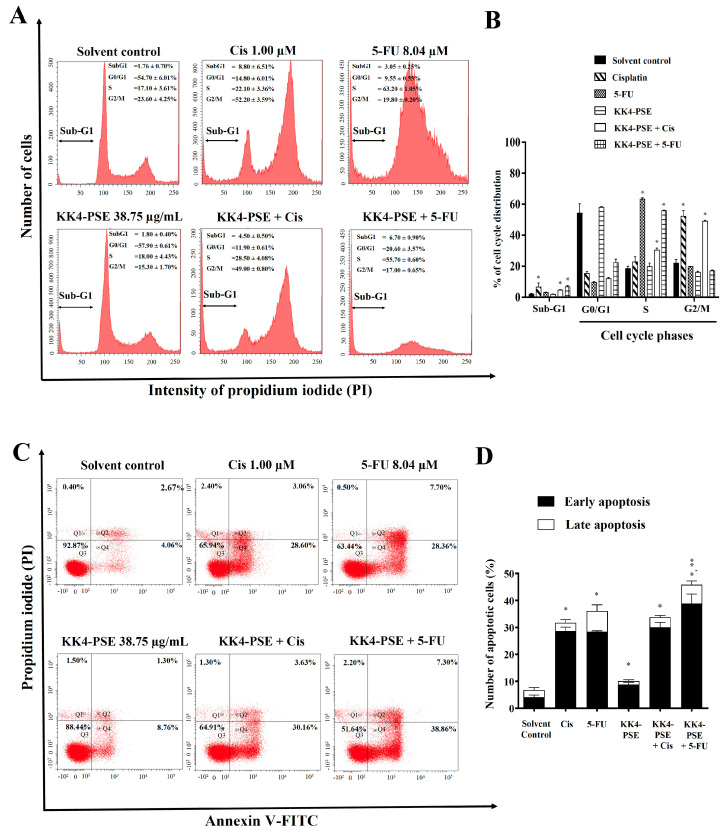
Effects of the combination treatments under the synergistic conditions on inductions of cell cycle arrest and apoptosis. (**A**) Representative DNA histograms of HeLa cells treated with IC_20_ sub-toxic doses (48 h exposure) of KK4-PSE, 5-FU, and cisplatin (Cis) either alone or in combination treatments are shown. (**B**) The percentages of cells at different cell cycle phases are shown as bar graphs of the mean from three independent experiments. (**C**) The representative dot plots represent the flow cytometry analysis of apoptosis induction in HeLa cells. (**D**) The bar graph shows the mean of the percentage of apoptotic cells from three independent experiments. HeLa cells were treated with solvent control (0.25% ethanol + 0.25% DMSO), cisplatin (1.00 µM), 5-FU (8.04 µM), and KK4-PSE (38.75 µg/mL), and the respective combination treatments for 48 h. * *p* < 0.05 and ** *p* < 0.05 indicate a significant difference compared with the solvent control and single agent treatments, respectively.

Based on the above results, the synergistic effect of KK4-PSE on HeLa cells was observed only in the 5-FU combination treatment. We further investigated whether the inductions of cell cycle arrest and apoptosis were related to the ability of KK4-PSE to induce cytotoxicity in combination treatments. The combination treatment of KK4-PSE with 5-FU at 48 h exposure caused increased sub-G1 (6.70 ± 0.90%) and S phase arrest (55.70 ± 0.60%), while the combination treatment of KK4-PSE with cisplatin caused increased sub-G1 (4.50 ± 0.50%) and G2/M phase arrest (49.00 ± 0.80%), compared with the KK4-PSE single drug treatment (sub-G1 = 1.80 ± 0.40%, S = 18.00 ± 4.43%, G2/M = 15.30 ± 1.70%) (Figure 2A,B). The combined 5-FU and KK4-PSE treatment caused an increase in the sub-G1 population compared with the single treatments of 5-FU and KK4-PSE (Figure 2B). 

Treatment with KK4-PSE alone enhanced apoptosis in HeLa cells compared with the solvent control treatment but KK4-PSE in combination with cisplatin did not cause a significant increase in apoptotic cells compared with the treatment with cisplatin alone (Figure 2C,D). In contrast, the combined 5-FU and KK4-PSE treatment under synergistic conditions caused enhanced apoptotic induction in HeLa cells (Figure 2C,D). This finding demonstrated that when 5-FU and KK4-PSE were used in combination, they greatly enhanced apoptosis in HeLa cells compared to the single agent treatments.

### 3.4. Effect of the Combination Treatments on Proteins Related to Apoptosis, ERK Signaling, and Acetylated Histone 

**Figure 3:**
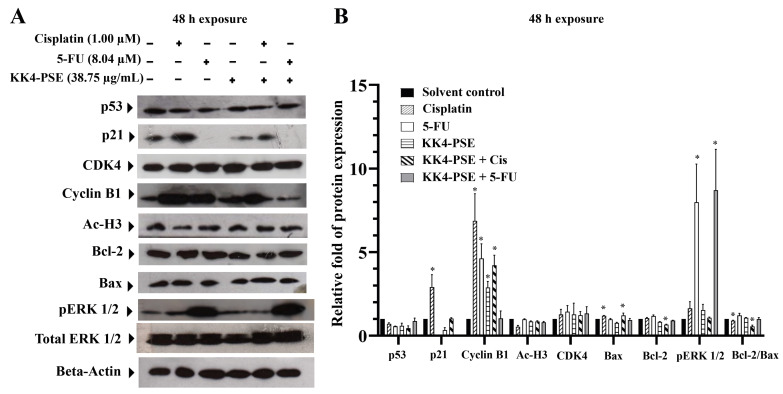
Effect of KK4-PSE in combination with cisplatin (Cis) or 5-FU on the levels of proteins involved in apoptosis and ERK signaling. HeLa cells were treated with the solvent control (0.25% ethanol + 0.25% DMSO), KK4-PSE (38.75 µg/mL), and Cis (1.00 µM) or 5-FU (8.04 µM) for single and combined agent treatments under the synergistic conditions (48 h exposure). (**A**) The representative protein bands from Western blot analysis are shown. Total ERK1/2 and β-actin were used as loading controls. (**B**) Bar graph represents mean ± SD of relative fold of protein expression from three independent experiments. “*” denotes a statistically significant difference (*p* < 0.05) compared with the solvent control treatment.

To elucidate the molecular mechanisms underlying the anticancer effects of the combination therapy, the expression levels of key proteins involved in apoptosis regulation (pro-apoptotic Bax and anti-apoptotic Bcl2), cell cycle control (cyclin B1), and ERK signaling (pERK1/2) were evaluated using Western blot analysis. In HeLa cells, the cisplatin treatments both alone and in combination with KK4-PSE significantly upregulated cyclin B1 protein expression compared with the solvent control treatment (Figure 3). The relative ratios of Bcl2/Bax were not significantly changed in the treatments with 5-FU both alone and in combination with KK4-PSE; however, the relative ratios of Bcl2/Bax were significantly decreased in the treatments with cisplatin both alone and in combination with KK4-PSE (Figure 3B). The combination of 5-FU and KK4-PSE significantly downregulated cyclin B1 compared to single agent treatments (Figure 3A,B). The upregulation of p-ERK was observed in 5-FU treatments both alone and in combination with KK4-PSE. Interestingly, 5-FU treatments significantly down-regulated the expression of p21 both alone and in combination with KK4-PSE (Figure 3A,B). The hyperacetylation of histone protein was not observed at the concentration of KK4-PSE used in this study. 

### 3.5. Antitumor Effect of KK4-PSE in Combination with 5-FU or Cisplatin on HeLa Xenograft Mice 

**Figure 4:**
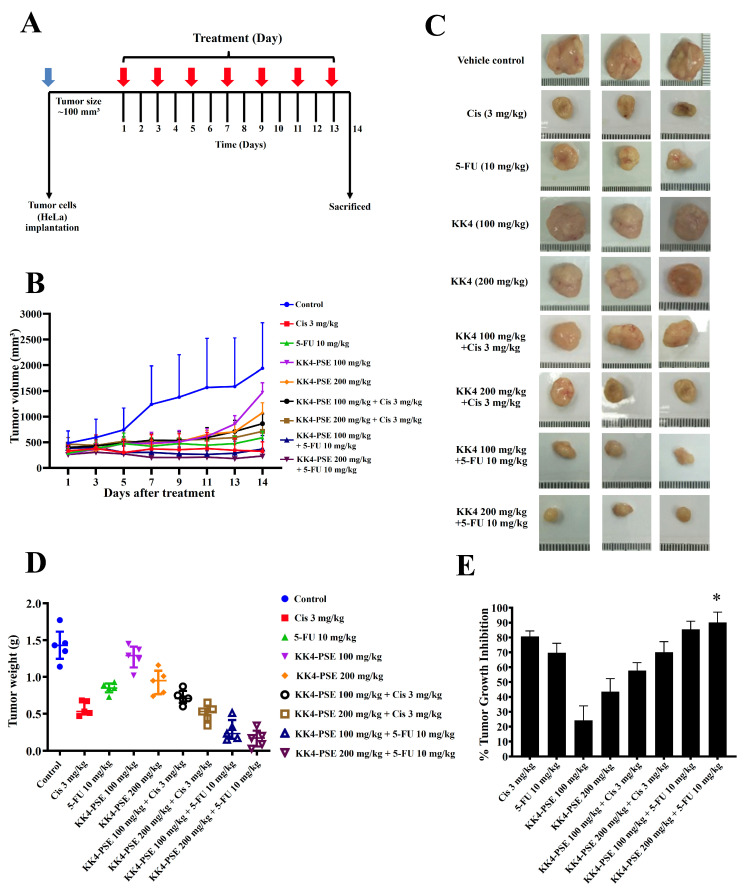
Effects of KK4-PSE (100 or 200 mg/kg), Cis (3 mg/kg), and 5-FU (10 mg/kg) alone and in combination on the HeLa mouse xenografts. (**A**) Experimental design of the administration of KK4-PSE, Cis, and 5-FU alone or in combination is shown. (**B**) Tumor volumes of HeLa-inoculated mice after treatments with KK4-PSE, Cis, and 5-FU alone or in combination are shown. (**C**) Representative photographs of the tumors and (**D**) tumor weights after surgical excision are shown. (**E**) The percentages of tumor growth inhibition (%TGI) compared with the control treatments are shown. “*” indicates a significant increase when compared with the single drug treatment (*p* < 0.05).

After implanting HeLa cells in female BALB/cAJcl-Nu/Nu mice for 14 days (the tumor volume reached ~100 mm^3^), the xenograft mice were intraperitoneally (i.p.) injected every other day for 14 days with normal saline solution as a vehicle control, 5-FU, cisplatin, and KK4-PSE both alone and in combination as depicted in Figure 4A. After drug administration, the length and width of each mouse’s tumor were measured using a digital Vernier caliper at each time point to calculate the tumor volume (Figure 4B). After the mice were euthanized in a CO₂ chamber, the tumors were dissected and photographed (Figure 4C). The groups of mice treated with individual agents, cisplatin (3 mg/kg), 5-FU (10 mg/kg), and KK4-PSE (100 and 200 mg/kg), exhibited a reduction in tumor volumes when compared with the vehicle control group (Figure 4B). Moreover, the groups treated with KK4-PSE 200 mg in combination with 5-FU showed a greater decrease in tumor volume when compared with the vehicle control group (Figure 4B). Among all groups, mice receiving KK4-PSE (100 and 200 mg/kg) in combination with 5-FU showed significantly greater tumor weight reductions compared with the control group (Figure 4D). In single drug treatments, the percentages denoting tumor growth inhibition (%TGI) in mice treated with cisplatin (3 mg/kg), 5-FU (10 mg/kg), KK4-PSE (100 mg/kg) and KK4-PSE (200 mg/kg) were 85.40 ± 6.09%, 69.69 ± 6.43%, 24.17 ± 9.82%, and 44.50 ± 10.03%, respectively, compared with the vehicle control group (Figure 4E). In mice subjected to combination treatments with cisplatin (3 mg/kg) and KK4-PSE at doses of 100 mg/kg and 200 mg/kg, the %TGI values were 56.81 ± 5.79% and 70.02 ± 7.20%, respectively (Figure 4E). In comparison, the mice treated with the combination of 5-FU (10 mg/kg) and KK4-PSE at doses of 100 mg/kg and 200 mg/kg exhibited %TGI values of 83.59 ± 8.85% and 90.06 ± 6.98%, respectively (Figure 4E). 

**Figure 5:**
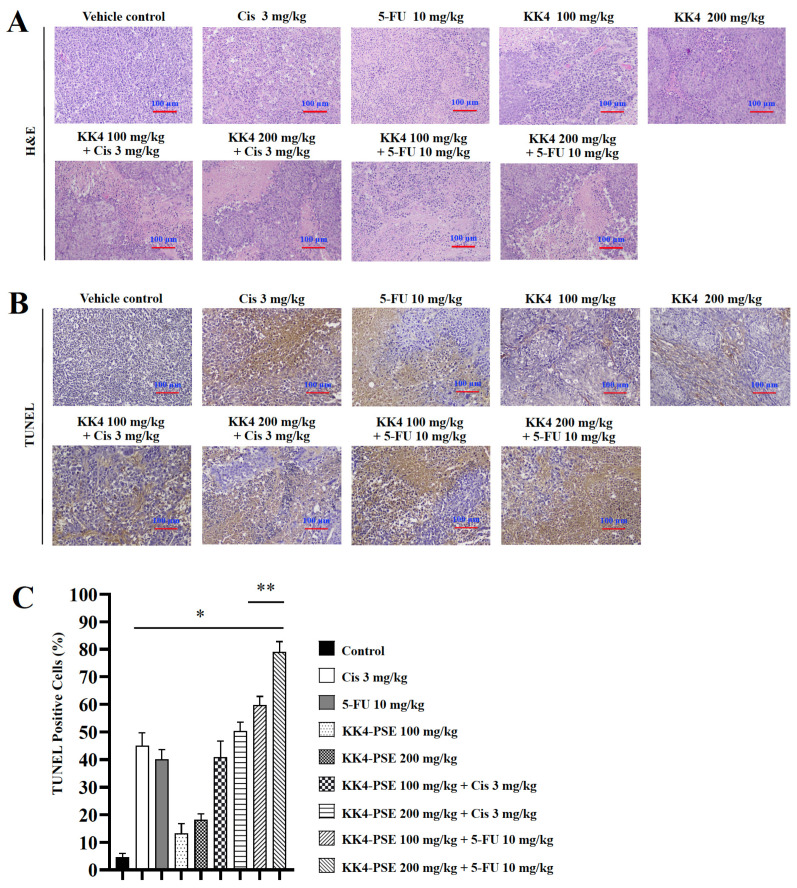
Effects of KK4-PSE, cisplatin, and 5-FU in single and combined agent treatments on HeLa cells-inoculated nude mice. (**A**) Hematoxylin and eosin staining (H&E) was used to analyze the histopathology of mouse tumor slices under a light microscope. (**B**) Representative tumor sections representing in situ apoptosis were analyzed by TUNEL staining. (**C**) Bar graph shows the mean percentage of TUNEL-positive cells as representing the level of apoptosis. “*” and “**” denote a statistically significant difference (*p* < 0.05) compared with the solvent control and single agent treatments, respectively. Scale bar = 100 μm.

Tumor sections from mice treated with the combined therapy exhibited features of apoptotic cells with nuclear condensation (Figure 5A). This observation aligned with the TUNEL assay results, where increased brownish cells signifying cellular apoptosis were predominantly observed in the treatment groups compared with the vehicle control group (Figure 5B,C). Notably, the 5-FU combination treatments with KK4-PSE (both 100 and 200 mg/kg) caused a significant increase of TUNEL-positive cells compared with the 5-FU alone treatment (Figure 5B,C). 

### 3.6. Toxicological Evaluation in Nude Mouse Xenograft Models 

Systemic toxicity of drugs to xenograft-bearing mice during treatment was evaluated by monitoring changes in body weight, organ weight, and histopathology of organs (liver, kidneys, and spleen). Baseline and final body weights are shown in Table 2. The body weights of mice treated with 100 and 200 mg/kg KK4-PSE alone were increased by 1.21% and 1.31%, respectively, compared with the control group. In contrast, mice treated with cisplatin, and 5-FU alone experienced weight decreases of 2.71% and 26.82%, respectively. The combination treatments of cisplatin with 100 and 200 mg/kg KK4-PSE caused a weight loss of 4.38% and 13.24%, respectively, while the combination treatment of 5-FU with 100 and 200 mg/kg KK4-PSE caused a weight loss of 16.53% and 23.33%, respectively. Furthermore, organ indices were calculated as the ratio of organ weight to body weight (Table 2). In mice treated with the combined KK4-PSE (200 mg/kg) and 5-FU (10 mg/kg), a significant increase in liver weight was observed when compared with the vehicle control. The kidney weights were significantly increased in mice treated with 5-FU in both alone and combination treatments compared with the vehicle control group. Conversely, both individual drug treatments and their combined treatment resulted in a significant reduction in spleen weight compared with the vehicle control group. 

**Figure 6:**
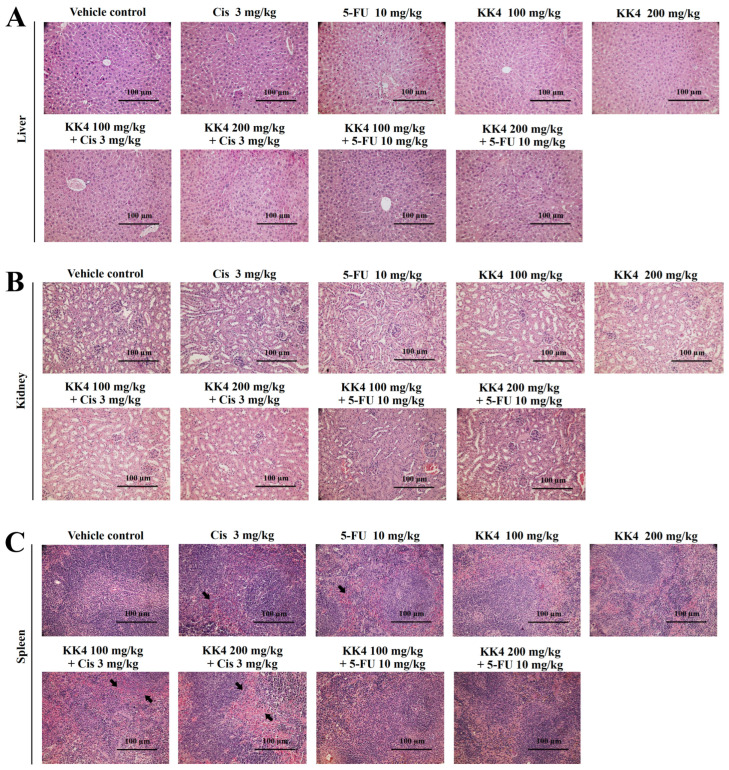
Histopathology of mouse organs. The tissue sections of (**A**) liver, (**B**) kidney, and (**C**) spleen were stained with hematoxylin and eosin and examined under a light microscope. The black arrow indicates spleen hyaloserositis that is often described as inflammation of the serous membranes (serositis) that cover tissues or organs. Scale bar = 100 μm.

Histological examination of liver and kidney tissues revealed no substantial variations between mice treated with individual therapies or their combinations compared with the vehicle control group (Figure 6A,B). These organs exhibited normal morphology without signs of significant drug-induced toxicity. In contrast, the spleens of mice treated with cisplatin, 5-FU, and cisplatin combined with KK4-PSE, displayed sparse necrotic and apoptotic cells, alongside vacuoles within the splenic corpuscles and periarterial lymphatic sheaths. These features are indicative of hyaloserositis, a pathological condition of the spleen (Figure 6C). Furthermore, treatment with individual drugs or their combinations with KK4-PSE resulted in some splenocytes exhibiting denser nuclei compared to the control group. 

## 4. Discussion

In this study, we further employed in vitro models to investigate the drug-interaction effects against HeLa cells and in vivo experiments to assess the antitumor efficacy and organ toxicity profiles in mouse models. The KK4-PSE exhibited potent antiproliferative activity against HeLa cells (IC_50_ = 41.32 ± 0.74 µg/mL) at 72 h exposure. This effect was significantly less pronounced in Vero cells (IC_50_ = 78.72 ± 2.83 µg/mL), suggesting a selective cytotoxicity towards cancer cells (Figure 1).

To quantitatively evaluate the drug interaction effects, the Chou–Talalay method was employed to calculate CI and DRI values [24,25]. These variables indicate if the interaction is synergistic, antagonistic, or additive, as previously defined [24,29]. In detail, these parameters determine whether the combined effect on cancer cells is additive (CI = 0.90–1.1), synergistic (CI < 0.90), or antagonistic (CI > 1.3) [24,29]. After 48 h of exposure, the combination of KK4-PSE and cisplatin exhibited an additive effect on HeLa cells (CI = 1.18 ± 0.06). Conversely, the KK4-PSE and 5-FU combination demonstrated a slight synergism at the same time point (CI = 0.49 ± 0.02) (Table 1). This synergism translated to a 3.19-fold and 21.78-fold reduction in the required doses of Cis and 5-FU, respectively, in HeLa cells after 48 h exposure. Notably, the 5-FU combination further facilitated dose reduction by 21.78-fold and 5.09-fold at 48 and 72 h, respectively. These findings suggest that KK4-PSE in combination with 5-FU exhibits greater potential for enhanced HeLa cell inhibition compared to the combination with cisplatin.

Combined treatment of KK4-PSE with either cisplatin or 5-FU potently suppressed HeLa cell proliferation through a dual mechanism: cell cycle arrest and apoptosis inductions. Treatment-specific cell cycle arrest was observed, with Cis inducing G2/M arrest and 5-FU targeting the S phase (Figure 2A,B). Consistent with our current findings, in previous studies, 5-FU triggered S-phase arrest in drug-resistant neck and head carcinoma cell lines (UM-SCC-23 and UM-SCC-2/WR) and in colon cancer cells [30,31]. Interestingly, 5-FU combinations can activate the thymidylate synthase (TS) gene [32]. This activation, however, appears to provide cancer cells with additional time to repair DNA damage induced by 5-FU, ultimately contributing to drug resistance [33,34]. Combinations of KK4-PSE with cisplatin/5-FU led to a significant increase in the sub-G1 population, indicating apoptosis induction (Figure 2A,B). Indeed, apoptosis analysis further confirmed and visualized this enhanced apoptosis (Figure 2C,D).

Several cancer cells were defeated by the synergistic effect of 5-FU and herb extract, which switched the manner of action from cell cycle disruption to increasing reactive oxygen species-mediated cell death [35,36]. In this study, our findings revealed that a combination treatment of cisplatin and KK4-PSE promoted cancer cell growth inhibition through up-regulating p21 and cyclin B1 (Figure 3). Cyclin B1 protein is suppressed by the p21 protein through preventing the activation of cyclin B1-cdc2 complexes, eventually resulting in G2/M phase cell cycle disruption [37]. Notably, the combination treatment of 5-FU and KK4-PSE caused the up-regulation of pERK1/2 (Figure 3). As with this finding, low-intensity DNA damage induced ERK activation, causing cell cycle arrest by phosphorylation of p53 [38]. Similarly, activating ERK1/2 can cause histone H3 phosphorylation by activating downstream histone kinases, resulting in oncotic cell death [39]. Furthermore, ERK activation is linked to cell death caused by ROS through signaling pathways downstream of p53 activation [40]. 

The BALB/cAJcl-Nu/Nu nude mouse HeLa xenograft model was used to investigate the empirical basis for the preclinical administration of 5-FU in combination with KK4-PSE. In this study, the KK4-PSE tumor suppression activity of in combination with 5-FU was significantly greater than that of cisplatin combination (Figure 4B–E). This is in accordance with our in vitro findings, according to which which KK4-PSE synergistically enhances the antiproliferative activity of 5-FU whether the interaction of KK4-PSE and cisplatin is additive or antagonistic (Table 1). There were no significant differences in tumor volumes between the combination treatments (5-FU + KK4-PSE) and 5-FU single drug treatment; however, the mean values of tumor volumes of the combination treatments (5-FU + KK4-PSE) were less than those of the 5-FU single drug treatment (Figure 4B). Nevertheless, significant differences in tumor weights and tumor growth inhibition ratios between the combination treatments (5-FU + KK4-PSE) and the 5-FU single drug treatment were observed (Figure 4D,E). Consistent with these findings, in the percentages of TUNEL-positive cells representing apoptotic-positive cells in the mouse tumors, significant differences between the combination treatments (5-FU + KK4-PSE) and the 5-FU single drug treatment were observed (Figure 5B,C). 

Toxicological evaluation revealed that nude mice treated both with 5-FU alone and in combination with KK4-PSE experienced slightly negative impacts on body weight gain following the intervention sessions (Table 2). Prolonged dosing of 5-FU has been reported to cause a significant hepatotoxicity as measured by biochemical and histopathological markers, in which total protein content (TPC) and overall liver weight were reduced in particular [41]. In this study, mice administered with 5-FU combined with high dose-KK4-PSE exhibited a significant increase in liver weight when compared with other treatment groups, suggesting that hepatotoxicity in mice may be relieved by the 5-FU combination treatment to minimize 5-FU toxicity. These results are in accordance with previous studies demonstrating that some herb extracts and phenolic compounds can reduce the hepatotoxicity of 5-FU by scavenging free radicals and lowering damage caused by ROS [42,43]. Furthermore, our data showed that KK4-PSE treatments, both alone and in combination with 5-FU, significantly increased the mouse kidney as well as liver weights (Table 2). The treatments with 5-FU alone or in combination with KK4-PSE significantly reduced spleen weight. However, the spleen hyaloserositis was significantly reduced in the 5-FU combination treatment with KK4-PSE when compared to the 5-FU alone treatment (Figure 6C), suggesting that KK4-PSE may also reduce spleen toxicity caused by 5-FU. Taken together, the KK4-PSE appeared to reduce the toxicities of 5-FU chemotherapy. However, clinical studies on the anticancer activity and the toxicity of the combined 5-FU and KK4-PSE are still required. 

The phenolic acid contents of KK4-PSE were partially identified in our previous study based on availability of phenolic acid standards [12]. That study indicated that *p*-hydroxybenzoic and *p*-coumaric acids were the most prevalent components in KK4-PSE. Furthermore, these phenolic components possessed histone deacetylase inhibitory activity against breast and cervical cancer cell lines [20]. Recent report revealed that peanut skin includes a variety of bioactive substances that may be divided into three categories: stilbenes, phenolic acids, and flavonoids, in which phenolic compounds are considered key active molecules with a wide variety of biological activity [44]. Peanut skin extracts have considerable amounts of phenolic compounds, including *p*-coumaric, cinnamic acids, ferulic, caffeic, chlorogenic, and quinic acids [45,46]. Gaafar et al. also reported that a crude extract from peanut skin exhibited cytotoxic effects on HCT116 (colon cancer), HepG2 (liver cancer), and MCF-7 (breast cancer) cells [11]. Furthermore, polyphenolic extract of peanut skin showed no cytotoxicity on normal epithelial cells and human peripheral blood cells [13]. Resveratrol found in peanut skin exhibits anticancer activity through a mechanism that involves disrupting the cellular signaling pathway and triggering apoptosis [47]. In addition, other promising active compounds in KK4-PSE will also be studied further before starting the clinical trial.

## 5. Conclusions

The combination treatment of 5-FU with KK4-PSE revealed synergistic anticancer effects against the human cervical cancer cell line. These synergistic effects may underpin a dose reduction of 5-FU by 5.09 and 21.78 folds at 48 h and 72 h exposures, respectively, against HeLa cells at 50% inhibition of cell proliferation. The up-regulation of p-ERK1/2, S phase cell cycle arrest, and apoptosis induction may be the key mechanisms for the synergistic interaction of 5-FU and KK4-PSE in cervical cancer (HeLa) cells. Furthermore, the combination treatment of 5-FU and KK4-PSE was less toxic to the non-cancer Vero cells in vitro than single agent treatment. In the mouse HeLa xenograft model, KK4-PSE also improved the antitumor growth of 5-FU via apoptosis induction. The 5-FU treatment in HeLa-inoculated nude mice caused toxicity in the spleen but not in the kidneys or liver, while the KK4-PSE appeared to reduce the toxicities of 5-FU chemotherapy. In short, KK4-PSE exhibits anticancer activity against cervical cancer cells both in vitro and in mouse xenograft models.

## Figures and Tables

**Table 1:** CI and DRI of the combination treatments between KK4-PSE, cisplatin, and 5-FU against HeLa cells.

Exposure Times	IC_50_ of KK4-PSE (µg/mL)		IC_20_ of Cis (µM)	IC_20_ of 5-FU (µM)	CI	DRI		
	Alone	Combination				Cis	5-FU	KK4-PSE
24 h	79.43 ± 0.54	43.37 ± 1.66	3.30 ± 0.14	-	1.12 ± 0.03	2.09	-	1.56
48 h	55.55 ± 1.57	45.48 ± 0.29	1.00 ± 0.17	-	1.18 ± 0.06	3.19	-	1.16
72 h	41.32 ± 0.74	35.83 ± 1.18	0.31 ± 0.07	-	1.02 ± 0.03	7.30	-	1.14
24 h	79.43 ± 0.54	31.79 ± 1.64	-	45.09 ± 3.64	N/D	-	N/D	2.13
48 h	55.55 ± 1.57	23.36 ± 0.23	-	8.04 ± 1.48	0.49 ± 0.02	-	21.78	2.26
72 h	41.32 ± 0.74	16.57 ± 1.34	-	1.50 ± 0.11	0.60 ± 0.03	-	5.09	2.49

KK4-PSE: Valencia KK4-type peanut skin extract; Cis: cisplatin; 5-FU: 5-fluorouracil. Antagonism: CI > 1.3; moderate antagonism: CI = 1.1–1.3; additive effect: CI = 0.9–1.1; slight synergism: CI = 0.8–0.9; moderate synergism: CI = 0.6–0.8; synergism: CI = 0.4–0.6; strong synergism: CI = 0.2–0.4. N/D = not determined.

**Table 2:** Body weight, % body weight change (%BWC), and relative organ weight of mice in the vehicle control and treated groups.

Groups	Initial Body Weight (g)	Final Body Weight (g)	%BWC	Organ Index (g/100 g Body Weight)		
				Liver	Kidney	Spleen
Vehicle control	23.52 ± 1.96	23.53 ± 1.45	0.04	6.27 ± 0.60	0.97 ± 0.09	1.60 ± 1.68
Cisplatin 3 mg/kg	23.61 ± 1.03	22.97 ± 1.23	−2.71	5.54 ± 1.88	0.80 ± 0.21 *	0.35 ± 0.18 *
5-FU 10 mg/kg	21.85 ± 1.12 *	15.99 ± 1.48 *	−26.82	7.37 ± 0.48	1.18 ± 0.10 *	0.28 ± 0.03 *
KK4-PSE 100 mg/kg	19.87 ± 1.36 *	20.11 ± 1.65 *	1.21	6.93 ± 0.58	1.04 ± 0.07	0.68 ± 0.17 *
KK4-PSE 200 mg/kg	19.80 ± 1.01 *	20.06 ± 0.78 *	1.31	7.13 ± 0.91	1.07 ± 0.08	0.76 ± 0.27 *
KK4-PSE 100 mg/kg + Cis	22.17 ± 1.05	21.20 ± 1.44	−4.38	6.98 ± 0.06	0.93 ± 0.05	0.71 ± 0.23 *
KK4-PSE 200 mg/kg + Cis	22.74 ± 1.55	19.73 ± 2.41 *	−13.24	7.02 ± 0.94	0.87 ± 0.06	0.70 ± 0.32 *
KK4-PSE 100 mg/kg + 5-FU	21.35 ± 1.10 *	17.82 ± 3.88 *	−16.53	7.17 ± 1.01	1.12 ± 0.10 *	0.32 ± 0.16 *
KK4-PSE 200 mg/kg + 5-FU	22.97 ± 0.30	17.61 ± 1.63 *	−23.33	7.98 ± 0.46 *	1.19 ± 0.07 *	0.21 ± 0.08 *

Data are expressed as mean ± S.D. (n = 5). Cis: Cisplatin; 5-FU: 5-fluorouracil; KK4-PSE: KK4 peanut skin ethanolic extract. “*” denotes a statistically significant difference (*p* < 0.05) compared with the vehicle control group.

## Data Availability

The original contributions presented in the study are included in the article, further inquiries can be directed to the corresponding author.
